# Volume Quantification of Acute Infratentorial Hemorrhage with Computed Tomography: Validation of the Formula 1/2ABC and 2/3SH

**DOI:** 10.1371/journal.pone.0062286

**Published:** 2013-04-24

**Authors:** Wanlin Yang, Yulan Feng, Yunyun Zhang, Jing Yan, Yi Fu, Shengdi Chen

**Affiliations:** 1 Department of Neurology and Institute of Neurology, Rui Jin Hospital, School of Medicine, Shanghai Jiao Tong University, Shanghai, China; 2 Department of Neurology, Minhang Central Hospital, Shanghai, China; 3 Department of Neurology, Yueyang Hospital Affiliated to Traditional Medical University, Shanghai, China; University of Jaén, Spain

## Abstract

**Objective:**

To compare the accuracy of formula 1/2ABC with 2/3SH on volume estimation for hypertensive infratentorial hematoma.

**Methods:**

One hundred and forty-seven CT scans diagnosed as hypertensive infratentorial hemorrhage were reviewed. Based on the shape, hematomas were categorized as regular or irregular. Multilobular was defined as a special shape of irregular. Hematoma volume was calculated employing computer-assisted volumetric analysis (CAVA), 1/2ABC and 2/3SH, respectively.

**Results:**

The correlation coefficients between 1/2ABC (or 2/3SH) and CAVA were greater than 0.900 in all subgroups. There were neither significant differences in absolute values of volume deviation nor percentage deviation between 1/2ABC and 2/3SH for regular hemorrhage (*P*>0.05). While for cerebellar, brainstem and irregular hemorrhages, the absolute values of volume deviation and percentage deviation by formula 1/2ABC were greater than 2/3SH (*P*<0.05). 1/2ABC and 2/3SH underestimated hematoma volume each by 10% and 5% for cerebellar hemorrhage, 14% and 9% for brainstem hemorrhage, 19% and 16% for regular hemorrhage, 9% and 3% for irregular hemorrhage, respectively. In addition, for the multilobular hemorrhage, 1/2ABC underestimated the volume by 9% while 2/3SH overestimated it by 2%.

**Conclusions:**

For regular hemorrhage volume calculation, the accuracy of 2/3SH is similar to 1/2ABC. While for cerebellar, brainstem or irregular hemorrhages (including multilobular), 2/3SH is more accurate than 1/2ABC.

## Introduction

Intracerebral hemorrhage (ICH) is one of the most serious life-threatening and crippling cerebrovascular diseases characterized by acute onset, rapid progression and high mortality rate. Spontaneous infratentorial hemorrhage mainly includes spontaneous cerebellar hemorrhage and brainstem hemorrhage. Spontaneous cerebellar hemorrhage occurs most often in the region of dentate nucleus, constituting 5 to 10% among all ICH cases with an incidence of about 1 in 33,000 people per year and a mortality rate ranging from 20% to 75% in different regions and age groups despite surgical treatments [1], [2], [3]. Should brainstem compression occur, the mortality rate might be up to 100% [1], [3]. Spontaneous brainstem hemorrhage accounts for 5 to 10% of all ICH cases, and occurs most frequently in the territory of pontine with an incidence of about 2 to 4 in 100,000 per year and a mortality rate ranging from 40% to 50% in different researches [4], [5]. Posterior cranial fossa has far less space than supratentorial region,accommodating cerebellar and brainstem. Furthermore, it houses several important headquarters in medulla oblongata including the cardiac, respiratory, vomiting, and vasomotor centers. Therefore, when a hematoma occurs in posterior cranial fossa, sudden increased intracranial pressure may lead to cerebellar tonsillar herniation and respiratory & circulatory failure, which might be fatal without timely identification or prompt treatment.

The hematoma volume is a convenient and reliable independent predictor for prognosis of ICH [6], [7]. ICH-Score, ICH-GS, and some other clinical grading scales for ICH prognosis, are all based on hematoma volume estimate [7], [8]. Some researchers recommend surgeries for cerebellar hemorrhage if volume >15 ml or maximal transverse diameter >3.0cm [9], [10], [11], [12], [13]. Furthermore, hematoma volume can also predict the prognosis of pediatric spontaneous intracerebral hemorrhage [14], [15], [16]. Therefore, a simple yet reliable estimation technique for hematoma volume must be very helpful for rapid prognosis and an appropriate treatment, especially for those who are potentially fit for emergency surgical intervention.

Computer-assisted volumetric analysis (CAVA) was widely considered the gold standard for estimation of hematoma volume [17], [18]. Although this technique is not difficult to manipulate, the calculating is extremely time-consuming and cumbersome without computer, which makes it inapplicable as a clinical routine [19], [20]. Kwak et al firstly introduced the formula 1/2ABC for estimation of ICH volume in 1983 which derived from volume calculation of ellipsoid [21]. It assumed that the shape of ICH compared to an idealized ellipsoid. It has been widely applied in clinical analysis and treatments internationally due to its simplicity, practicability and accuracy [14], [20], [22]. However, as it often overestimated or underestimated the volume of irregular shaped hemorrhage, researchers began to question its accuracy [20], [23], [24], [25], [26]. Therefore, Zhao et al introduced an updated formula 2/3SH which evolved from the bulk formula of ellipsoid, and proved that it was simpler and more accurate than formula 1/2ABC, 1/3ABC and Tada [27], [28].

More attention so far was put on supratentorial hemorrhages, few data were specifically designed for infratentorial hemorrhage. Hence, our objective was to compare the accuracy of formula 1/2ABC with 2/3SH in a cohort of infratentorial hemorrhage patients with different lesion locations and shapes.

## Materials and Methods

### Inclusion and Exclusion Criteria

The study was approved by the ethics committee of Rui Jin Hospital Affiliated to Shanghai Jiao Tong University School of Medicine. This retrospective study only involved patients’ CT scans which were stored in PACS system. Based on the advice from the committee, our study did not have problems of informed consent. We retrospectively screened and analysed the medical records of 147 patients from three hospitals in Shanghai (Rui Jin Hospital, Minhang Central Hospital and Yueyang Hospital) from 2001 to 2011. All patients collected in this retrospective study were diagnosed spontaneous cerebellar hemorrhage or brainstem hemorrhage, with hypertension. The neurologist and neuroradiologist made a consensus on the diagnosis. The first cranial CT scan was performed within 24 hours after the onset of symptoms. The exclusion criteria are as followed: (1) subdural, epidural and subarachnoid hemorrhage; (2) ICH attributed to brain tumors; (3) traumatic hemorrhage; (4) isolated intraventricular hemorrhage; (5) recurrent intracerebral hemorrhage; (6) ICH due to arteriovenous malformation.

### Patient Groups

According to the location, the hematomas were categorized into two groups: (1) cerebellar hemorrhage; (2) brainstem hemorrhage. Based on shape of the layer of maximum hemorrhage area, hematomas were also divided into three groups: (1) regular, including round or ellipsoid with smooth and clear margins; (2) irregular; (3) multilobular was subdivided as a kind of special irregular shape [20]. Furthermore, cerebellar hemorrhages with maximal transverse diameter >3.0cm were screened out.

### Imaging

147 cranial non-contrasted CT scans (64-slice, GE, America) were obtained and the slice thickness was 5 or 10 mm for infratentorial hemorrhage. Recorded the following data: (1) shape and location of hematoma; (2) the maximal longitudinal and transverse diameters of the largest slice of hematoma (cm); (3) area of hemorrhage in each slice (cm^2^); (4) slice thickness (cm). Two physicians independently recorded the above data and calculated volume values (CAVA, formula 1/2ABC and 2/3SH). Moreover, a mutual decision was made in cases of disagreement. The readers were blinded of clinical data. Finally, the average value of two observers was calculated for further statistical analysis. Therefore, no measurement could bias the other.

### Estimation Techniques

In CAVA method, the margins of hematoma in each slice were outlined by hand and the corresponding areas were automatically generated by computer software. In particular, the edema around the hemorrhage was excluded. The volume of ICH was achieved by summing up the volumes of each slice (area×slice thickness). In formula 1/2ABC, A is the maximal cross-sectional diameter in the largest slice of hemorrhage, B is the largest diameter perpendicular to A at the same slice, and C is the height of hematoma (number of slices with hemorrhage multiplied by slice thickness). In formula 2/3SH, S is the area of largest axial hemorrhage slice, while H is the height of hematoma derived from the slice thickness times the number of slices.

### Statistical Analysis

Statistical analysis was performed using SPSS software package (SPSS18.0). Intra-class correlation coefficient (ICC) was calculated to evaluate the interrater reliability. A value of >0.8 relates to excellent reliability, while 0 means complete inconsistency. In this study, the ICC for infratentorial hemorrhage volume calculated by two observers was 0.955 which means an excellent reliability.

To compare the accuracy of formula 1/2ABC with 2/3SH, the absolute values of volume deviation ([formula–gold standard ml) and percentage deviation ([formula–gold standard]/gold standard %) were calculated for further statistical analysis, respectively. Correlation coefficients were performed with Spearman test to determine the correlation of the measuring techniques. After the distributions of all data were in turn evaluated by Shapiro-Wilk test. Data was expressed as median ± interquartile range for non-normal distribution. Hematoma volumes calculated by three techniques were compared using the Kruskal-Wallis test. The absolute values of volume deviation (ml) and percentage deviation (%) of formula 1/2ABC and 2/3SH were compared using Wilcoxon matched-pairs signed ranks test, respectively. Categorical variables were compared with Fisher’s exact test. A value of *P*<0.05 was considered statistically significant.

## Results

From 2001 to 2011, 147 patients with infratentorial hemorrhage were collected in this study. The average age was 68.07±13.21 years, 88 males (59.9%) and 59 females (40.1%). The most common shape was irregular (96 cases, 65.3%), which was almost double the regular (51 cases, 34.7%). Within the former group, 21 cases were multilobular (14.3%). 84 patients with cerebellar hemorrhage (57.1%) contained 35 cases of regular shaped hemorrhage (41.7%) and 49 cases of irregular shaped hemorrhage (58.3%). Meanwhile, 63 patients with brainstem hemorrhage (42.9%), mainly occurred in pontine, included 16 cases of regular shaped hemorrhage (25.4%) and 47 cases of irregular shaped hemorrhage (74.6%). Hemorrhage shape was statistically different between cerebellar and brainstem hemorrhage (*P*<0.05). More irregular shaped hemorrhages occurred in the latter (Table 1).

**Table 1:** Characteristics of 147 cases included.

Characteristic	Gold standard	1/2ABC	2/3SH
Age(years)	68.1±13.2	–	–
Gender			
Male	88(59.9%)	–	–
Female	59(40.1%)	–	–
Hematoma volume(ml)	4.83(1.93, 9.52)	3.66(1.46, 8.50)	4.31(1.47, 9.28)
Infratentorial hematomas volume misclassified (15 ml)	–	5	3
Location			
Cerebellar hematoma	84(57.1%)	–	–
Regular	35(41.7%)		
Irregular	49(58.3%)		
Brainstem hematoma	63(42.9%)	–	–
Regular	16(25.4%)		
Irregular	47(74.6%)		
Shape			
Regular	51(34.7%)	–	–
Irregular	96(65.3%)	–	–
Multilobular	21(14.3%)	–	–

Volumes are presented as median +/− interquartile range.

**Figure 1:**
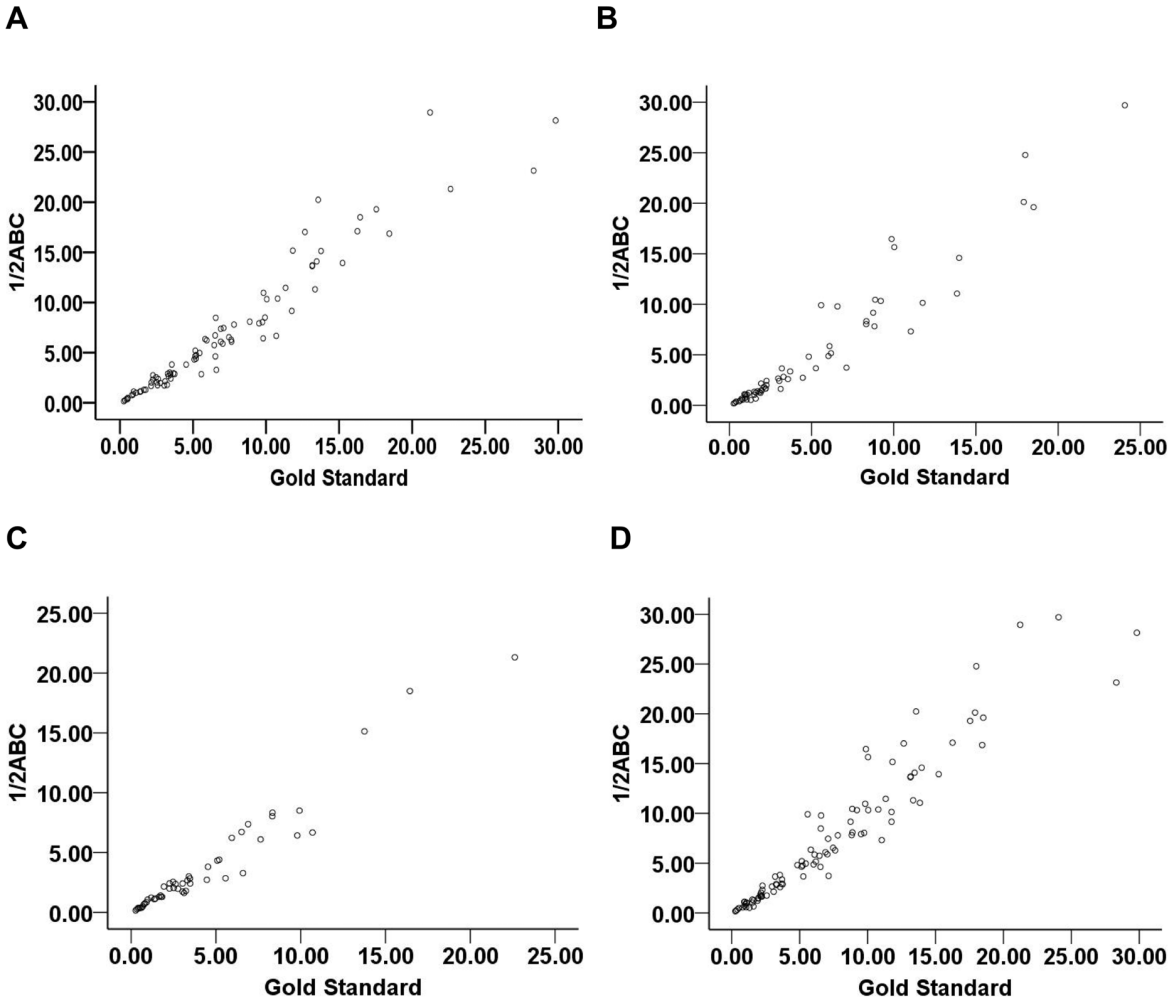
The correlation coefficients of formula 1/2ABC with CAVA (gold standard). (A) for cerebellar hemorrhage, the correlation coefficient was 0.980; (B) for brainstem hemorrhage, the correlation coefficient was 0.979; (C) for regular shaped hemorrhage, the correlation coefficient was 0.974; (D) for irregular shaped hemorrhage, the correlation coefficient was 0.976. CAVA, computer-assisted volumetric analysis.

**Figure 2:**
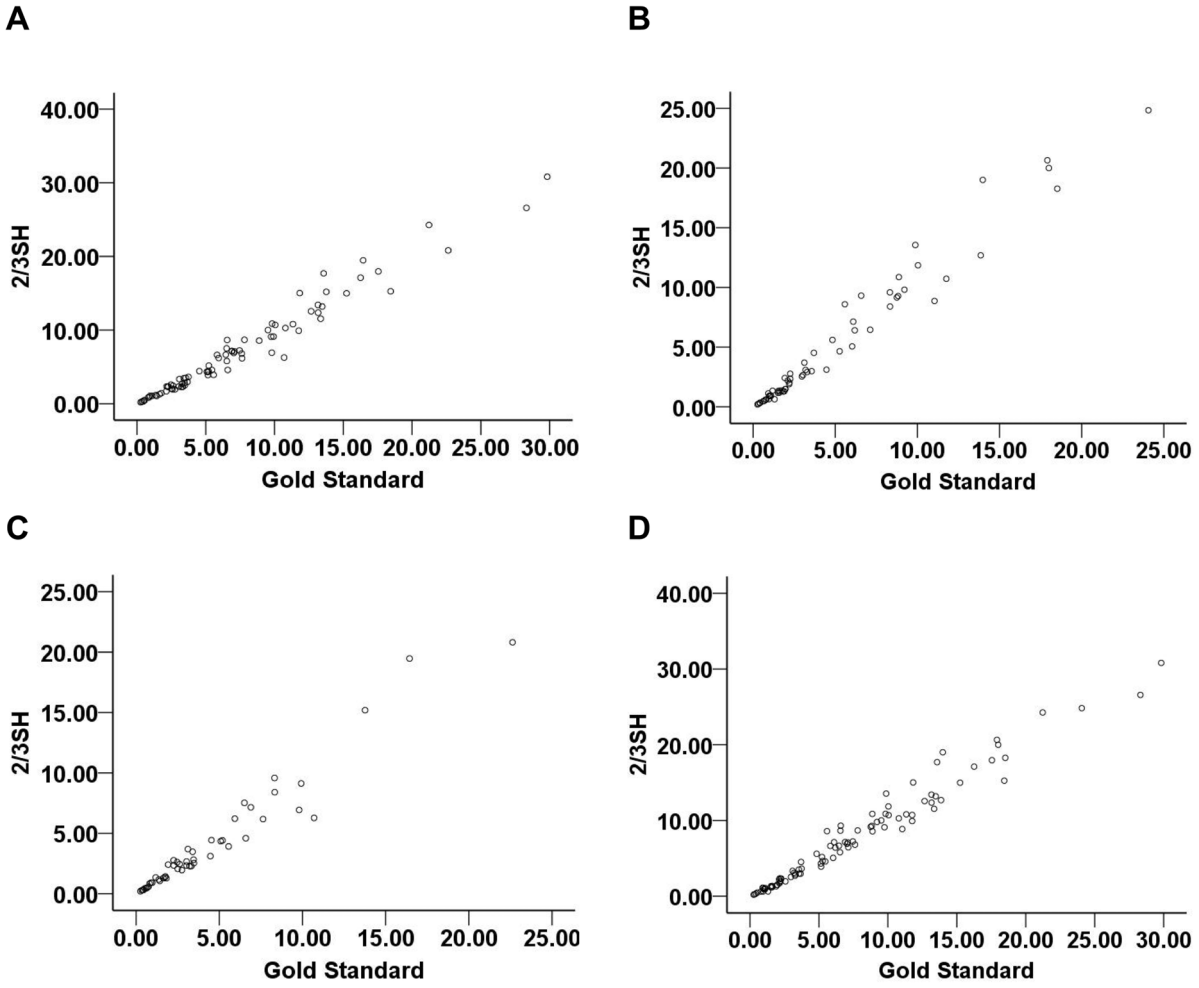
The correlation coefficients of formula 2/3SH with CAVA (gold standard). (A) for cerebellar hemorrhage, the correlation coefficient was 0.983; (B) for brainstem hemorrhage, the correlation coefficient was 0.988; (C) for regular shaped hemorrhage, the correlation coefficient was 0.972; (D) for irregular shaped hemorrhage, the correlation coefficient was 0.987. CAVA, computer-assisted volumetric analysis.

The correlation coefficients of formula 1/2ABC and 2/3SH with gold standard were described respectively below: 0.980 and 0.983 for cerebellar hemorrhage, 0.979 and 0.988 for brainstem hemorrhage, 0.974 and 0.972 for regular shaped hemorrhage, 0.976 and 0.987 for irregular shaped hemorrhage. Evidently, two formulas were all excellently comparable to the gold standard (Fig. 1, Fig. 2).

**Figure 3:**
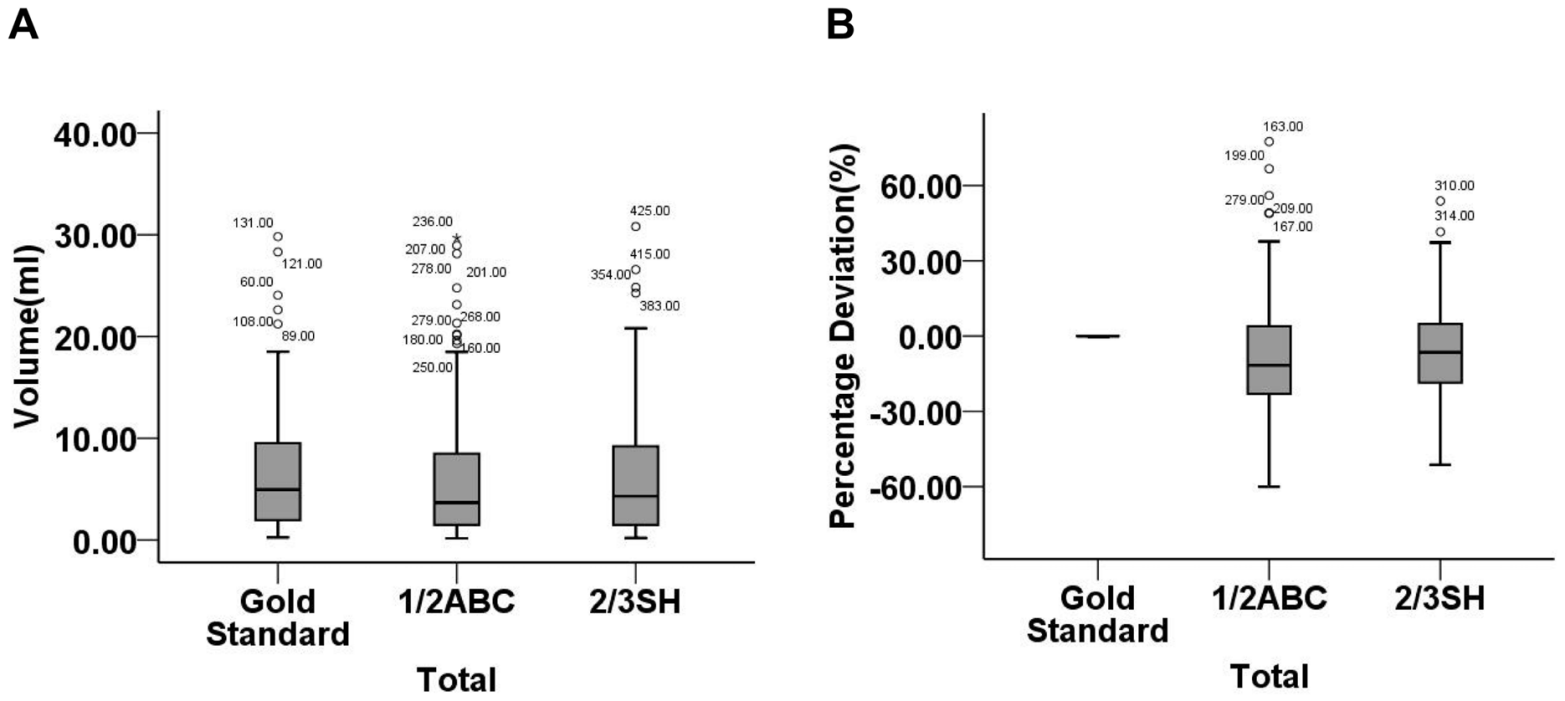
Hematoma volumes (ml) and percentage deviations (%) for total 147 infratentorial hemorrhages calculated by gold standard, formula 1/2ABC and 2/3SH. (A) the comparison of three methods in hematoma volume was statistically indistinguishable (*P* = 0.700); (B) formula 1/2ABC and 2/3SH underestimated hematoma volume by −11.59(−23.07, 4.13)% and −6.51(−18.58, 4.88)%, respectively. CAVA, computer-assisted volumetric analysis.

For 147 infratentorial hemorrhages, the comparison of three methods in hematoma volume was statistically indistinguishable (*P* = 0.700). The absolute values of volume deviation and percentage deviation calculated by formula 1/2ABC were greater than 2/3SH (*P*<0.05). Furthermore, compared with gold standard, hematoma volume was underestimated by an average of 12% and 7% for formula 1/2ABC and 2/3SH, respectively (Table 2, Fig. 3).

**Table 2:** Hematoma volumes, absolute values of volume deviation (ml) and percentage deviation (%) for gold standard, 1/2ABC and 2/3SH in total 147 patients.

N	Methods	Hematoma volume (ml)	Absolute value of volumedeviation (ml)	Absolute value of percentage deviation (%)
147	Gold standard	4.83(1.93,9.52)	–	–
	1/2ABC	3.66(1.46,8.50)	0.55(0.25,1.42)	16.68(8.98,27.95)
	2/3SH	4.31(1.47,9.28)	0.48(0.18,0.95)	14.32(5.27,22.63)
	Chi-square	0.713	–	–
	Z	–	−4.457	−4.612
	*P*-value	0.700	0.000	0.000

Values are presented as median +/− interquartile range.

**Figure 4:**
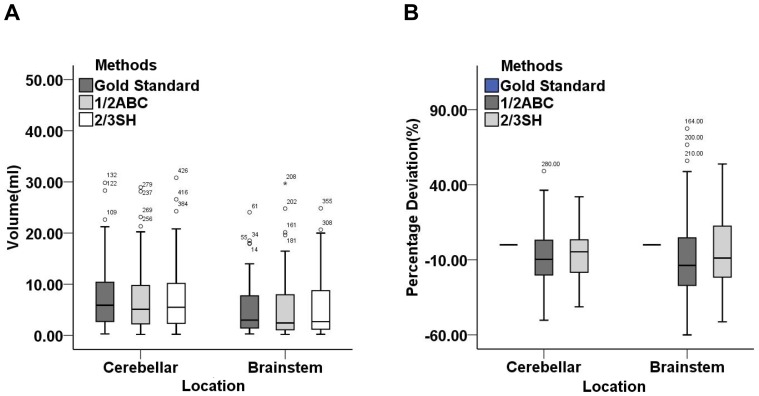
Hematoma volumes (ml) and percentage deviations (%) for cerebellar and brainstem hemorrhages calculated by gold standard, formula 1/2ABC and 2/3SH. (A) the comparison of three methods in hematoma volume was statistically indistinguishable (*P*>0.05); (B) formula 1/2ABC and 2/3SH underestimated hematoma volume by −10.34(−20.11, 3.10)% and −4.64(18.31, 3.36)% for cerebellar hemorrhage, −13.69(−27.15, 4.78)% and −8.80(−21.74, 13.88)% for brainstem hemorrhage, respectively.

For cerebellar and brainstem hemorrhages, hematoma volumes estimated were not significantly different between two subgroups by three methods (*P*>0.05). The absolute values of volume deviation and percentage deviation calculated by formula 1/2ABC were greater than 2/3SH too (*P*<0.05). Formula 1/2ABC and 2/3SH underestimated hematoma volume by 10% & 5% for cerebellar hemorrhage and by 14% & 9% for brainstem hemorrhage, respectively (Table 3, Fig. 4).

**Table 3:** Hematoma volumes, absolute values of volume deviation (ml) and percentage deviation (%) for gold standard, 1/2ABC and 2/3SH in different locations.

Methods	Hematoma volume (ml)		Absolute value of volume deviation (ml)		Absolute value of percentagedeviation (%)	
	Cerebellar	Brainstem	Cerebellar	Brainstem	Cerebellar	Brainstem
Gold standard	5.88(2.65,10.54)	2.97(1.31,8.33)	–	–	–	–
1/2ABC	5.08(2.18,10.04)	2.41(1.07,8.03)	0.71(0.29,1.43)	0.48(0.22,1.15)	15.04(7.45,22.84)	19.10(11.45,31.96)
2/3SH	5.49(2.33,10.21)	2.66(1.17,8.87)	0.54(0.18,0.98)	0.43(0.18,0.82)	10.93(3.58,18.94)	16.21(9.43,24.03)
Chi-square	0.511	0.426	–	–	–	–
Z	–	–	−3.43	−2.862	−3.791	−2.718
*P*-value	0.744	0.808	0.001	0.004	0.000	0.007

Values are presented as median +/− interquartile range.

**Figure 5:**
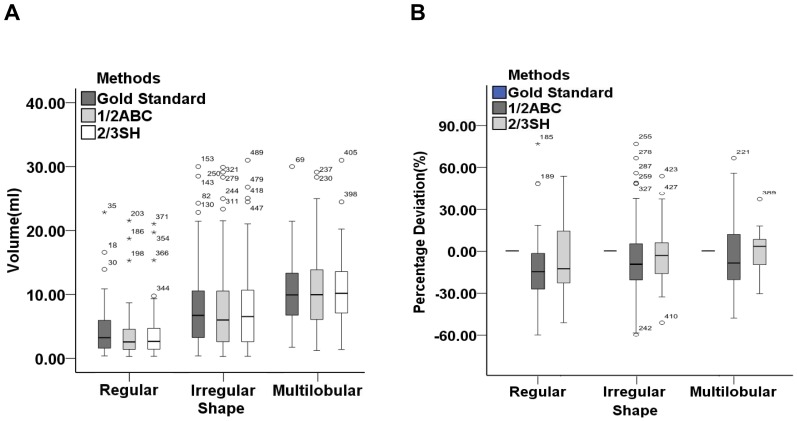
Hematoma volumes (ml) and percentage deviations (%) for regular, irregular and multilobular hemorrhages calculated by gold standard, formula 1/2ABC and 2/3SH. (A) the comparison of three methods in hematoma volume was statistically indistinguishable (*P*>0.05); (B) formula 1/2ABC and 2/3SH underestimated hematoma volume by −18.51(−28.20, −5.80)% and −15.56(−25.85, 3.38)% for regular hemorrhage, −9.26(−20.75, 5.19)% and −3.23(−15.78, 6.17)% for irregular hemorrhage, respectively. However, for the multilobular shaped hemorrhage, the formula 1/2ABC underestimated by −8.53(−16.73, 4.25)% and 2/3SH overestimated only by 1.96(−6.37, 9.68)%.

For regular, irregular and multilobular shaped infratentorial hemorrhage, hematoma volumes were not significant different in three subgroups measured by three techniques, respectively (*P*>0.05). There were neither significant differences in the absolute values of volume deviation nor percentage deviation between formula 1/2ABC and 2/3SH for regular shaped hemorrhage (*P*>0.05). However, the two above-mentioned absolute values calculated by formula 1/2ABC were greater than 2/3SH for irregular and multilobular shaped infratentorial hemorrhage (*P*<0.05). The formula 1/2ABC and 2/3SH underestimated hematoma volume by 19% & 16% for regular shaped hemorrhage and 9% & 3% for irregular shaped hemorrhage, respectively. However, for the multilobular shaped hemorrhage, the formula 1/2ABC underestimated the volume by nearly 9% while 2/3SH overestimated it by 2% (Table 4, Fig. 5).

**Table 4:** Hematoma volumes, absolute values of volume deviation (ml) and percentage deviation (%) for gold standard, 1/2ABC and 2/3SH in different shapes.

Methods	Hematoma volume (ml)			Absolute value of volume deviation (ml)			Absolute value of percentage deviation (%)		
	Regular	Irregular	Multinodular	Regular	Irregular	Multinodular	Regular	Irregular	Multinodular
Gold standard	3.03(1.37,5.94)	6.14(2.20,10.98)	9.75(6.50,13.57)	–	–	–	–	–	–
1/2ABC	2.37(1.15,4.40)	5.47(1.77,10.83)	9.79(5.82,14.15)	0.40(0.16,1.29)	0.69(0.35,1.60)	1.03(0.55,1.69)	18.51(9.71,28.20)	15.88(8.86,27.11)	16.22(5.00,30.07)
2/3SH	2.45(1.17,4.59)	6.54(2.07,10.85)	10.01(6.78,14.34)	0.37(0.14,0.95)	0.50(0.20,0.94)	0.52(0.26,2.05)	18.51(8.02,25.85)	11.25(4.70,19.55)	6.62(3.56,15.78)
Chi-square	1.262	0.126	0.097	–	–	–	–	–	–
Z	–	–	–	−1.219	−4.403	−2.172	−1.519	−4.542	−2.381
*P*-value	0.532	0.939	0.953	0.223	0.000	0.030	0.129	0.000	0.017

Values are presented as median +/− interquartile range.

In this paper, there were 9 cerebellar hemorrhages with volume greater than 15 ml calculated by gold standard. Compared with the gold standard, formula 1/2ABC misclassified 5 cases (4 false-positive results, 1 false-negative result), while 2/3SH misclassified 3 cases (3 false-positive results, 0 false-negative result). Although there was no statistical significance between formula 1/2ABC and 2/3SH, the former might be more likely to cause inaccuracy in the calculation.

Thirty-four cases of cerebellar hemorrhage with maximal transverse diameter greater than 3 cm were screened out. The comparison of three techniques in hematoma volume was no statistical significance (*P*>0.05). The absolute values of volume deviation and percentage deviation calculated by formula 1/2ABC were larger than 2/3SH (*P*<0.05) (Table 5).

**Table 5:** Hematoma volumes, absolute values of volume deviation (ml) and percentage deviation (%) for gold standard, 1/2ABC and 2/3SH in 34 cases with maximum transverse diameter>3cm.

N	Methods	Hematoma volume (ml)	Absolute value of volume deviation (ml)	Absolute value of percentage deviation (%)
34	Gold standard	11.83 (8.88, 16.35)	–	–
	1/2ABC	11.46(8.07, 17.80)	1.37 (0.67, 3.07)	11.56 (5.43, 21.81)
	2/3SH	12.36 (8.89, 17.83)	0.87(0.43,2.42)	6.62(3.43, 17.84)
	Chi-square	0.204	–	–
	Z	–	−2.421	−2.297
	*P*-value	0.903	0.015	0.022

Values are presented as median +/− interquartile range.

## Discussion

By analyzing the 147 CT images with infratentorial hemorrhage, the major findings are as below: (1) overall, the formula 1/2ABC and 2/3SH all didn’t significantly differ from gold standard, the three techniques had excellent consistency; (2) for cerebellar and brainstem hemorrhage, formula 2/3SH might be more accurate than 1/2ABC. They all had a tendency to underestimate hematoma volume in all locations; (3) for regular shaped hemorrhage, the formula 2/3SH didn’t significantly differ from 1/2ABC. For irregular and multilobular shaped hemorrhage, 2/3SH might be much more precise than 1/2ABC. The formula 1/2ABC underestimated hematoma volume for all shapes. However, 2/3SH slightly overestimated the volume of multilobular shaped hemorrhage while underestimated other shaped hemorrhages; (4) the formula 2/3SH might be more accurate than 1/2ABC for cerebellar hemorrhages with maximal transverse diameter larger than 3cm. However, 2/3SH didn’t significantly differ from 1/2ABC for cerebellar hemorrhages with volume greater than 15 ml.

The hematoma volume has become a convenient and reliable independent predictor for prognosis of ICH patients [14], [27]. Several studies had validated that, when transverse diameter >2 cm or volume >5 ml, 30-day mortality rate of patient with brainstem hemorrhage was very high and suggested surgical treatment such as stereotactic surgery or microsurgery [29], [30], [31], [32]. For cerebellar hemorrhage, surgery was also recommended when volume >15 ml or maximal transverse diameter >3 cm [9], [10], [11], [12], [13]. Hence, a simple yet reliable estimation technique for hematoma volume is very important in clinical practice. In contrast, an inaccurate estimation of hematoma volume may result in wrong decisions and serious consequences.

Kothari et al validated the formula 1/2ABC could accurately calculated hematoma volume in less than 1 min [22]. Furthermore, its effectiveness and convenience have been repeatedly demonstrated for the measurement of acute parenchymal, subdural, epidural and even pediatric intracerebral hematoma volume [14], [15], [16], [19], [20]. Now formula 1/2ABC has been widely used for bedside calculation of hematoma volume in hospital. However, some researchers doubted its accuracy since it was based on the assumption that a hematoma could be compared to an idealized ellipsoid. As hematoma shapes were not always regular, theoretically, it should be more applicable for regular shaped hematoma than irregular shaped hematoma. Gebel et al found that 1/2ABC overestimated the hematoma volume by 8.5% [19]. Huttner et al calculated the volume of warfarin-related hematoma and founded that the formula 1/2ABC significantly overestimated the volumes of multinodular or separated by almost 32%; Furthermore, they founded that measurement error was gradually increased when hematoma volume was decreased and the adjustment formula 1/3ABC was much more accurate than 1/2ABC for irregular shaped hematoma [20]. Divani et al demonstrated that 1/2ABC overestimated the hematoma volume by about 8% [23]. Wang et al [24] validated that, formula 1/2ABC overall overestimated hematoma volume by 29.3% compared with CAVA. Therefore, Zhao et al introduced a new formula 2/3SH for estimation of hematoma volume [27], [28].

Our results showed that 1/2ABC had a tendency to underestimate the hematoma volume compared with CAVA, which was consistent with the researches of Sheth and Freeman [25], [26]. Importantly, 1/2ABC underestimated volume and might result in an overoptimistic prognosis, especially for those potentially fit for emergency surgical treatment, which might lead to inappropriate and premature abandonment of surgical protocols. If formula 2/3SH turned out to be more accurate than 1/2ABC, no matter formula 1/2ABC overestimated or underestimated the volume, it would be very helpful for clinician to predict prognosis and enact protocol for patients more accurately. Our results demonstrated that, compared with formula 1/2ABC, 2/3SH could more accurately estimate the volume of irregular shaped hematoma. 2/3SH underestimated the volume by 3% for irregular and overestimated by 2% for the volume of multilobular. Because when hematoma shape changed, the maximum length and width might be invariable, while the area of largest axial slice of hematoma (S) would change as well [27], [28]. Hence, the area would be more representative of a hematoma than the two above-mentioned diameters. Furthermore, some researchers hold the idea that irregular shape could independently predict ICH expansion, which might be the major cause of morbidity and mortality [33], [34], [35], [36]. Therefore, in clinical practice, formula 2/3SH might compensate for the deficiencies inherent in formula 1/2ABC to some extent and assisted clinicians in enacting appropriate treatment protocol to improve prognosis of patients. Our study validates volume as an imaging biomarker on acute hemorrhage management.

There are some limitations in this paper: (1) this was a retrospective study. We only collected one hundred and forty-seven patients which might not entirely reflect the characteristics of infratentorial hemorrhage as subdural, epidural, subarachnoid, traumatic, isolated intraventricular and recurrent infratentorial hemorrhages, ICH due to brain tumors were excluded; (2) the volume of regular hematoma was underestimated more seriously than irregular shape by formula 1/2ABC and 2/3SH, which was inconsistent with previous researches. The hematoma volumes of 147 patients were generally small. Only 9 cerebellar hematoma volumes calculated by gold standard were greater than 15 ml in this study. Meanwhile, the volume of irregular hematoma was roughly twice as regular hematoma (6.14 ml vs 3.03 ml, *P*<0.05). A slight change in volume for small hematoma might lead to wide fluctuations in percentage deviation [20], [27], [28]. This meant that formula 1/2ABC might not suitable for small hematoma. Further analysis of large hematoma (including supratentorial and infratentorial) can further determine the value of 2/3SH method; (3) slice thickness of CT scans was 5 or 10 mm for infratentorial hemorrhage in this paper. Further studies with a homogeneous slice thickness could confirm the results.

### Conclusions

Although the prognosis of patients with cerebral hemorrhage can be affected by many factors, the hematoma volume is one of the important bases for treatment (including surgery) in clinical practice. A simple yet reliable estimation technique for hematoma volume can provide important evidences for decision of surgical or conservative treatment. In summary, CAVA, formula 1/2ABC and 2/3SH do not have essential differences. The formula 2/3SH doesn’t significantly differ from 1/2ABC on regular shaped hematoma. For cerebellar, brainstem and irregular (including multilobular) shaped hematomas, formula 2/3SH may be more accurate than 1/2ABC. Although 1/2ABC has been widely used clinically, it still needs some further improvements and developments. 2/3SH is a simple, rapid, and accurate estimation technique for hematoma volume, which may compensate for deficiencies of formula 1/2ABC to some extent. In the future, because of smaller infratentorial hematomas, large-scale prospective study should be carried out to analyze the influence of formula 1/2ABC and 2/3SH for prognosis and treatment decision.
